# Non-Destructive Evaluation for Corrosion Monitoring in Concrete: A Review and Capability of Acoustic Emission Technique

**DOI:** 10.3390/s150819069

**Published:** 2015-08-05

**Authors:** Ahmad Zaki, Hwa Kian Chai, Dimitrios G. Aggelis, Ninel Alver

**Affiliations:** 1 Department of Civil Engineering, Faculty of Engineering, University of Malaya, Kuala Lumpur 50603, Malaysia; E-Mail: ahmad.zaki@siswa.um.edu.my; 2 Department of Mechanics of Materials and Constructions, Vrije Universiteit Brussel, Pleinlaan 2, Brussels 1050, Belgium; E-Mail: Dimitrios.Aggelis@vub.ac.be; 3 Department of Civil Engineering, Faculty of Engineering, Ege University, Bornova, Izmir 35100, Turkey; E-Mail: ninel.alver@ege.edu.tr

**Keywords:** reinforced concrete, corrosion, non-destructive testing, acoustic emission

## Abstract

Corrosion of reinforced concrete (RC) structures has been one of the major causes of structural failure. Early detection of the corrosion process could help limit the location and the extent of necessary repairs or replacement, as well as reduce the cost associated with rehabilitation work. Non-destructive testing (NDT) methods have been found to be useful for *in-situ* evaluation of steel corrosion in RC, where the effect of steel corrosion and the integrity of the concrete structure can be assessed effectively. A complementary study of NDT methods for the investigation of corrosion is presented here. In this paper, acoustic emission (AE) effectively detects the corrosion of concrete structures at an early stage. The capability of the AE technique to detect corrosion occurring in real-time makes it a strong candidate for serving as an efficient NDT method, giving it an advantage over other NDT methods.

## 1. Introduction

Corrosion of steel reinforcement is a global problem that leads to deterioration of RC structures [1]. Damage induced by steel corrosion usually requires proper repair, followed by maintenance [2,3]. It is reported that the costs of repair and maintenance of corroded structures exceed billions of dollars per year [4]. The costs vary depending on the condition of the concrete structures, including the cause of damage, degree of damage, and effect of damage on structural behavior [5]. A reliable inspection method is required at an early stage before the functionality of the RC structure is seriously damaged due to steel corrosion. The inspection method usually provides information about the current condition of the RC structures, so that their future performance can be predicted. Furthermore, the inspection method is almost a prerequisite for efficient and cost effective rehabilitation of existing RC structures. The inspection should be done without damaging the RC structures, both new and old ones.

The NDT methods are of many inspection methods for corrosion monitoring in RC structures. For new structures, the principal applications of NDT methods are likely to be quality control of the concrete conditions, while in old structures, the methods are expected to provide needed feedback on monitoring, detection, and identification of damage [6]. This paper aims to give a brief review of the NDT methods for monitoring and evaluating steel corrosion in RC structures, followed by a discussion and review of a technique based on AE developed to achieve the same objectives. Due to its principles of application, it is considered that AE has the capability of monitoring the steel corrosion in RC structures at an early stage.

## 2. NDT Methods for Corrosion Monitoring

Recently, various methods have been implemented for corrosion monitoring in RC structures. They are classified into six main categories as follows: visual inspection, electrochemical methods (*i.e.*, open circuit potential (OCP) monitoring, resistivity method, polarization resistance, galvanostatic pulse method (GPM), electrochemical noise (EN)), elastic wave methods (*i.e.*, ultrasonic pulse velocity (UPV), acoustic emission (AE), and impact echo (IE)), electromagnetic (EM) methods (*i.e.*, ground penetrating radar (GPR)), optical sensing methods (*i.e.*, fiber Bragg grating (FBG)), and infrared thermography (IRT). Visual inspection, OCP, polarization resistance, and other electrochemical methods are more commonly used for corrosion monitoring in RC structures.

### 2.1. Visual Inspection

Visual inspection is a regular inspection method to assess corrosion damage on the surface of concrete structures. The appearance of the corroded area often provides valuable insight into the cause and the extent of corrosion. However, the method is very dependent on the inspector’s experience. In addition, visual inspection is limited in its effectiveness to detect surface discontinuities due to steel corrosion and the unseen corrosion is difficult to spot [7,8,9,10].

### 2.2. Electrochemical Methods

**Figure 1:**
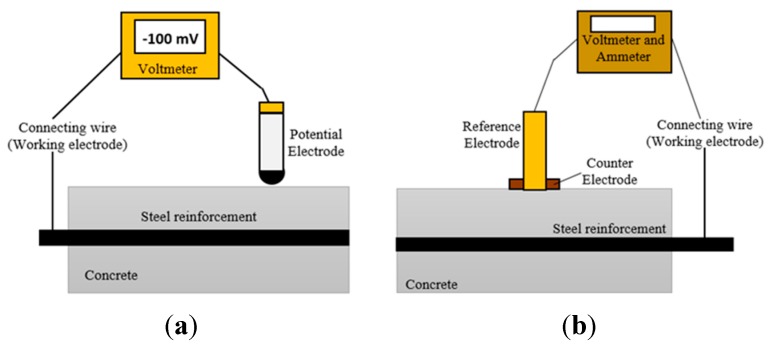

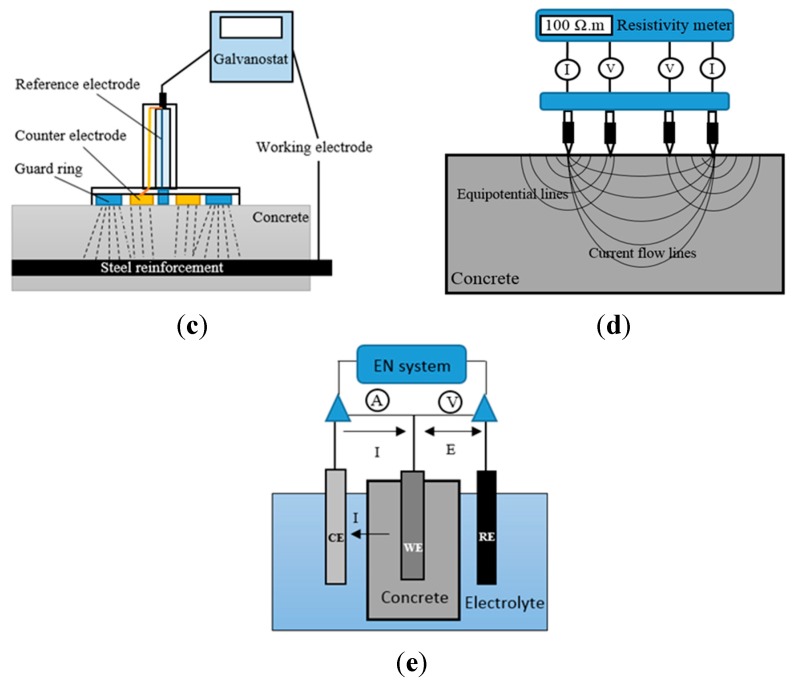
Principle of electrochemical methods: (**a**) open circuit potential monitoring (OCP) (Reproduced and modified from [7]); (**b**) polarization resistance (Reproduced and modified from [23]); (**c**) galvanostatic pulse method (GPM) (Reproduced and modified from [37]); (**d**) resistivity method (Reproduced and modified from [36]); and (**e**) electrochemical noise (EN) (Reproduced and modified from [38]).

Electrochemical methods are by far the most suitable for corrosion monitoring in RC structures. Electrochemical methods, in general, can provide fast and reliable information on the probability of corrosion, the corrosion rate of steel reinforcement, and the resistivity of the concrete structure [11,12,13,14,15,16,17,18,19,20,21,22]. Electrochemical methods are related to the interrelation of the electrical and the chemical effects. An electrochemical system measures the potential and current of oxidation and reduction reactions [23,24,25,26,27,28]. The principles of the five electrochemical methods are shown in Figure 1. 

In the open circuit potential (OCP) monitoring, one of the electrochemical methods, the electrical potential (in mV or V) between a steel reinforcement and a reference electrode (*i.e*., a copper/copper sulfate cell), in contact with the concrete surface, is measured [29,30,31,32]. The principle of OCP method is shown in Figure 1a. The OCP provides information pertaining to the probability and potential level of corrosion in RC structures [7,9]. Table 1 gives the potential ranges for different corrosion conditions of steel inside the reinforced concrete. The method is often unconvincing in terms of interpretation because the measurement depends on the condition of the RC structure. Moisture levels and amount of chloride concentration can affect the potential readings and give erroneous results [7,33]. The changes in moisture content (*i.e.,* wet condition of the concrete surface) lead to a shift in potential values so they become more negative value (*i.e.,* a shift of 100 mV was found on a bridge deck measured in the dry condition and the wet condition after rainfalls). The potential gradients and the local potential do not change; differences were found only in the magnitude of potential gradients. In a structure with a high chloride concentration, there is usually low resistivity in the concrete. The readings are often mismatched when there is low resistivity in the concrete, but it has readings with high passive potential. This is due the fact that chlorides are transported more easily in lower resistivity of concrete, high contents of chlorides depassivated the steel reinforcement, and active potentials were measured [7].

On the other hand, the polarization resistance method is commonly used for measuring the corrosion rate in RC structures. The method records the current generated and consumed by anodic and cathodic reactions. The change in potential during reactions is known as polarization, which is used to evaluate the steel corrosion. However, this electrochemical method has some limitations, such as the method assumes uniform corrosion while pitting corrosion is a highly probable form of steel corrosion in concrete, which might lead to misleading results. In addition, the area of steel measured in concrete is not precisely known which creates some errors in the polarization resistance calculations. Another error in the polarization resistance method is the IR drop introduced by high resistive medium and high separation of the reference electrode from the steel reinforcement of the RC structures [34,35,36]. Figure 1b shows the principal of polarization resistance method.

The other practical method for measuring the corrosion rate is GPM. This generally involves impressing a small amplitude, short time anodic current pulse, to be applied galvanostatically on the steel reinforcement from the external counter electrode over the concrete surface [39,40]. The anodic current is usually in the range of 5 to 100 μA and the typical pulse duration is between 5 and 30 s [19]. The steel reinforcement is anodically polarized and there is a resulting change in the electrochemical potential. The potential is recorded by a reference electrode (usually in the center of the counter electrode) as a function of polarization response. Figure 1c shows a schematic setup for the GPM test. When the constant current *I*_app_ is applied to the system, the polarized potential of reinforcement (*η*_t_), at given time t can be expressed as [39]:

*η*_t_ = *I*app[*R*_ct_[1−exp (−t / *R*_ct_ × *C*_dl_))] + *R*_Ω_] (1)

where *R*_ct_ is polarization resistance, *C*_dl_ is double layer capacitance, and *R*_Ω_ is ohmic resistance.

The GPM results are much closer to the corrosion rate produced by the gravimetric method than those produced by polarization resistance, electrochemical impedance spectroscopy (EIS), the Tafel extrapolation method (TEM), and the harmonic analysis method (HAM) [13,14,19,41,42]. Uncertainty about the polarized area of steel reinforcement is one of the major sources of error when measuring the corrosion rate of steel reinforcement in RC structures affected by the electrical signal from the counter electrode and the non-uniform current distribution of the steel reinforcement [43].

The resistivity method is another electrochemical method, which relies upon the principle that corrosion is an electrochemical process. An ionic current must pass between the anode and cathode areas for corrosion monitoring of RC structures [44]. The resistivity is an indirect indication of active corrosion of the steel reinforcement [45,46,47]. The corrosion process will be slower if the resistivity of the concrete is high. The resistivity of concrete exposed to chloride indicates the risk of early corrosion damage, because low resistivity is always associated with rapid chloride penetration [48]. Concrete resistivity is generally measured using the Wenner four probe method, as illustrated in Figure 1d. However, this method has limitations due to the heterogeneities (*i.e*., steel reinforcement, resistivity layers, cracks, and large aggregates), which would influence the shape of the electric lines in the concrete. In addition, the low distance of the electrode probe (less than a few tens of centimeters) and weather conditions (*i.e*., wet, temperature, and humidity) influence the resistivity, thus having a huge influence on the results [49,50].

On the other hand, the electrochemical noise (EN) is an emerging technique for monitoring the mechanisms and estimating the rate of corrosion in concrete structures. Electrochemical noise is used to describe fluctuations in the potential and current generated by corrosion reactions [51,52]. The phenomenon whereby the potential of the electrodes fluctuates due to current vibration is also referred to as noise [53]. The current vibration of electrodes is due to fluctuation of the oxide-reduction reaction on the surfaces of the electrodes [38]. A noise source is located within the probable corroding area. A noise signal is transformed from the time domain to the frequency domain displayed in the form of amplitude and frequency based on the fast Fourier transform (FFT). The noise resistance (*Rn*), which is believed to be similar to the polarization resistance, is given by [54,55,56]:

*Rn* = σV/σI (2)

where σV is the standard deviation for the potential and σI, the standard deviation for the current obtained by statistical analysis of the noise data.

The main advantage of the EN technique is its lack of intrusiveness. It can avoid artificial disturbances to the system during measurement [57]. The EN measurement has been used to study the onset of localized corrosion [58,59]. However, only a few studies of EN for measuring corrosion in RC structures have been carried out [60,61]. Previous work using this technique on reinforcing bars has only studied the corrosion process of various metallic materials [62,63,64,65].

**Table 1:** Interpretation of corrosion activity of electrochemical methods.

Corrosion Activity	Potential Level (mV) [53]	Resistivity (Ω·m) [51]	Corrosion Rate	
			LPR (I_corr_ (A/cm^2^)) [39]	GPM (*R*_ct_ (kΩ·cm^2^)) [39]
Very High	-	-	10–100	0.25–2.5
High	<−350	<100	1.0–10	2.5–25
Moderate/Middle	−200 to −350	100–500	-	-
Low	>−200	500–1000	0.1–1	25–250
Negligible/passive	-	>1000	<0.1	>250

Table 1 provides a summary of the qualitative corrosion activity of the above electrochemical methods. However, there is no standardization for the evaluation of the corrosion activity by EN method. Therefore, most of the electrochemical methods require direct connection with the steel reinforcement in the RC structure as the electrode, which makes it an intrusive method [66]. It requires a localized breakdown in the concrete surface to provide the direct connection [67]. 

### 2.3. Electromagnetic (EM) Waves

Another NDT method of corrosion monitoring is ground penetrating radar (GPR), which is based on the propagation of an EM wave into an RC structure. Part of the EM wave is reflected back to the receiving antenna whenever it encounters an interface of two media with differing dielectric constants [68,69,70,71]. A GPR antenna receives direct and reflected waves, which are recorded as amplitude–time signals (*a*-scan) by the system. The direct wave signal (*S*_d_) represents the EM energy transmitted directly to the receiving antenna, and the reflected wave (*S*_r_) is the EM energy reflected from the steel reinforcement–concrete interface [70]. The propagation of EM waves depends on the corresponding dielectric permittivity of concrete, which is a quantity related to the ability of concrete to resist the flow of an electrical charge [69,72,73,74]. The permittivity in turn depends on the EM properties, which are influenced by temperature, moisture content, chloride content, pore structure, and deterioration (*i.e*., corrosion) [75]. 

**Figure 2:**
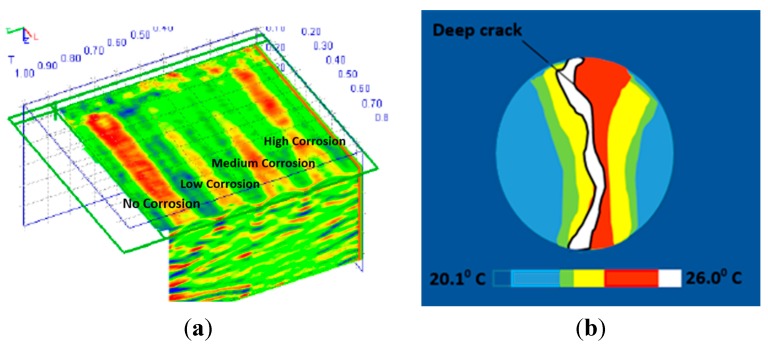
(**a**) 3D image of GPR of concrete slab with different level of steel corrosion, unit (m) (with permission from [68]) and (**b**) IRT image of concrete surface of cylinder, unit (°C) (Reproduced and modified from [89]).

Despite its capability, GPR only presents a qualitative assessment of the corrosion damage. Moreover, the presence of chloride content and corrosion products would attenuate the GPR waves [74,76,77,78], which decrease the amplitude of the wave and the average velocity of reflected wave [79,80,81] increasing the travel time of the wave [82,83]. In addition, the GPR method is carried out periodically to monitor the corrosion process in RC structures and cannot be used for real-time monitoring [66]. Figure 2a shows a 3D image of GPR data in an RC slab with four different degrees of corrosion (*i.e*., no corrosion, low corrosion, medium corrosion, and high corrosion from the left to the right side). 

### 2.4. Infrared Thermography (IRT)

As a sub-surface inspection method, Infrared thermography (IRT) has been found to be capable of detecting corrosion in RC structures. The method is based on the resulting perturbations in the heat transfer characteristics of concrete materials [84,85,86,87,88,89,90]. Temperature is one of the most common indicators of the structural health of RC structures. Cracks, alkali aggregate reaction (AAR), corrosion, and other deterioration forms could cause abnormal temperature distribution [86]. With the advent of newer generations of IR cameras, IRT is becoming a more accurate, reliable, and cost effective technique for corrosion monitoring in RC structures [88]. However, like GPR method, the interpretation of IRT is also qualitative, *i.e.*, a higher degree of steel corrosion results in stronger IR thermal distribution on the concrete surface [89,91,92,93,94], which would exhibit higher peaks of IR intensity and faster rates of heating [95,96], and hence increase the temperature of the concrete materials [92]. Figure 2b shows an IRT image of the temperature distribution of a 10% degree of corrosion of the concrete cylinder.

### 2.5. Optical Sensing Methods

The optical sensing technology of the NDT methods, the fiber Bragg grating (FBG) method, involves creating periodic variations in the refractive index of the core of an optical fiber [97]. When light is made to pass through the grating at a particular wavelength, called the Bragg wavelength, the light reflected by the varying zones of refractive indices would be in phase and amplified [98,99]. During corrosion, the formation of corrosion products less dense than steel increases the volume and diameter of the bars, leads to an increase in fiber strain, which is measured by a shift in the wavelength of the FBG. The extent of corrosion is quantitatively evaluated through the change in the wavelength of the FBG [100,101,102]. Although it has advantages such as linear reaction, small volume, high anti-erosion capability, and automatic signal transmission [103,104], the FBG method also has limitations in corrosion monitoring in RC structures. The method can only conduct localized inspection of steel corrosion in RC structures. It is expected that a distributed and long-gauge FBG technique will be developed to solve this problem; until then this method is less effective for corrosion monitoring in RC structures [105].

### 2.6. Elastic Wave Methods

In order to enhance reliability, elastic wave methods should be adopted to complete the assessment of steel corrosion in RC structures. Elastic wave methods are essential when estimating mechanical properties and inhomogeneous characterization due to steel corrosion in RC structures. Wave-based damage detection excites transient waves to propagate into the concrete structure using sensors since the waves are reactive to damage such as cracks, voids, and also corrosion products [106]. There are three major wave-based methods for corrosion monitoring in RC structures, *i.e*., ultrasonic pulse velocity (UPV), impact echo (IE), and acoustic emission (AE). 

**Figure 3:**
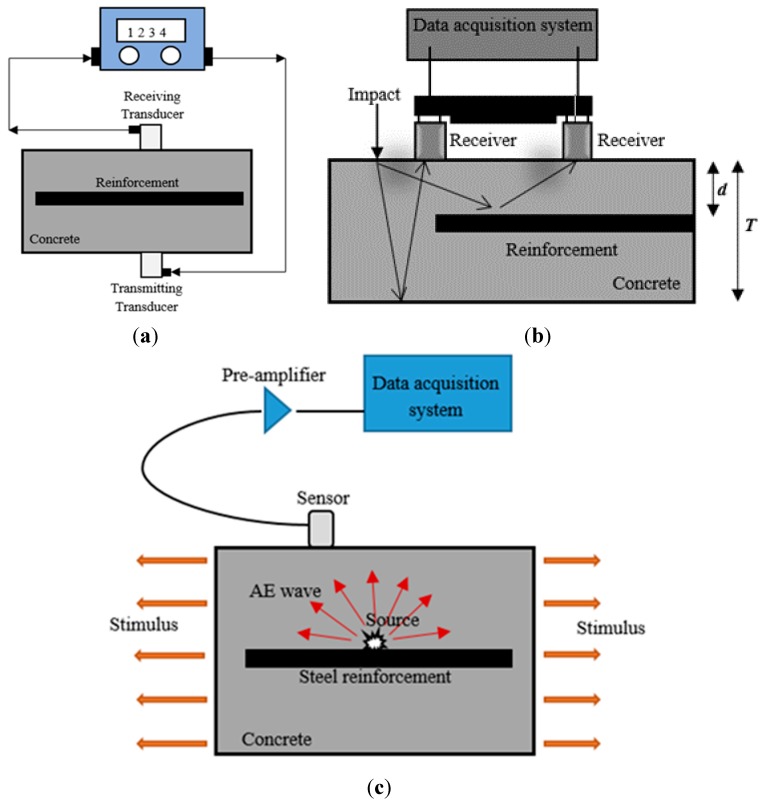
The principle of: (**a**) ultrasonic pulse velocity (Reproduced and modified from [120]); (**b**) impact echo (Reproduced and modified from [121]); and (**c**) acoustic emission (Reproduced and modified from [135]).

The UPV test is an NDT method that involves measuring the speed of sound through concrete in order to detect the condition of the concrete and the presence of steel corrosion [31,107,108,109,110,111,112] in chloride and oxide environments [112,113,114,115]. The principle of the UPV method is shown in Figure 3a. Ultrasonic waves can propagate a long distance along the steel reinforcement and have been found to be sensitive to the interface conditions between the steel reinforcement and the concrete [112,116]. Cracking due to steel corrosion results in wave attenuation and a decrease in UPV. It has been reported that the amplitude attenuation has good a correlation with the damage due to corrosion [31,117,118]. In addition, UPV as measured by the first wave peak could describe the process of steel corrosion. As the corrosion damage level increases, the relative variation for the first wave peak value of UPV first increases and then decreases [119]. This condition occurs because the corrosion products are increased during the process of steel corrosion, resulting in an increase in the delamination degree between the steel reinforcement and the concrete. As the reinforced concrete corrosion level increases, the pit on the steel becomes larger. This condition would lead to great reflection in the first wave energy, and the direct transmission wave energy would become low. The first wave peak value then decreased slowly. However, the method needs advanced study. The study could come from the use of surface and other, guided waves, with features other than longitudinal pulse velocity, and stemming from advanced signal processing techniques [120].

The stress wave method, IE, is employed to detect corrosion in structural elements using mechanical impact and then monitoring the displacement (d), detected by sensors placed on the concrete surface. The impulse is reflected by the arrival of reflections of the pulse from the crack and other internal defects under investigation [121,122], as shown in Figure 3b. In an early study by Liang and Su [123] more than a decade ago, the IE method was certainly able to detect the development of micro-cracks due to corrosion in RC blocks. In the latest studies, Samarkova *et al*. [124,125,126,127] have indicated that the dominant frequencies of the response signal are the main criteria used to detect the occurrence and position of steel corrosion in RC structures. However, only a number of limited studies have attempted to monitor steel corrosion in RC structures because the main application of the IE method is in the detection of voids and delamination. In addition, the proposed method is not as mature as electrochemical methods for corrosion monitoring.

Therefore, AE is considered a good complementary method to UPV and IE. The AE technique is a unique, non-invasive, and passive NDT method. AE is a class of phenomena whereby transient elastic waves (ultrasonic frequency range) from a localized source within a material and conversion of the waves into electrical signals through coupled piezoelectric sensors [128,129,130,131,132]. The sources of AE are deformation processes such as crack growth, void closure, plastic deformation, corrosion, and other material degradation. Localized energy release gives rise to elastic waves that are detected by sensors placed on the concrete surface [133,134]. The principle of AE is shown in Figure 3c. The AE technique is often capable of detecting corrosion in the early stages, so that an early warning can be given to allow for repair work before the structural RC element is seriously damaged and the functionality is lost due to steel corrosion. This study reviews comprehensively the capability of the AE technique in monitoring the corrosion activity and it discusses the distinct advantages of the AE technique in the following sections. Table 2 shows a summary of NDT methods for corrosion evaluation.

**Table 2:** Resume of NDT methods for corrosion evaluation.

No	NDT Methods	Principles	Advantages	Disadvantages	Corrosion Evaluation	Specific Equipment
**1**	**Electrochemical Method**					
	Open circuit potential (OCP) monitoring	Electrical potential value (in mV or V) is measured between steel reinforcement of RC and reference electrode (indicates corrosion potential of the steel inside RC).	The results are not in the form of equipotential contours, rather a single value that gives an indication of the steel condition.	Time consuming and need to be closed several hours during the inspection.	Potential level (mV or V)	Potential electrode, Voltmeter, and connecting wire (working electrode).
	Resistivity method	Resistivity (ρ) of RC, which the current can easily pass between anode and cathode areas of the concrete.	An easy, fast, portable, and inexpensive technique, which can be used for routine inspection.	Reinforcement in the test region can provide a “short-circuit” path and cause erroneous reduction in the measurement.	Resistivity (Ω·cm)	Current and potential electrodes, Voltmeter or resistivity unit, and insulated wire (working electrode).
	Polarization resistance	The change in potential during reactions (polarization) is recorded using an electrode plate on the concrete surface.	Short time for measurement and applies small perturbations that do not interfere with the existing electrochemical processes.	It takes time to obtain a full response because of the electrical capacitance across the steel and concrete interface. The voltage error introduced by IR drop in the concrete between working (steel rebars) and reference electrode.	Corrosion current (I*corr* (A/cm^2^))	Reference electrode, counter electrode, Voltmeter, Ammeter, and connecting wire (working electrode).
	Galvanostatic pulse method (GPM)	The anodic current pulse is applied galvanostatically on the steel reinforcement from counter electrode placed on the concrete surface.	A rapid device for determining the corrosion rate of steel reinforcement in RC, it enables display of corrosion rate, electrical resistance and potential value simultaneously.	Unstable reading due to parallel or crossing of the steel reinforcement, also cracks and delamination are often the reason for wrong readings.	Potential resistance *(R*_ct_ (kΩ·cm^2^))	Reference electrode, counter electrode, guard ring, and connecting wire (working electrode).
	Electrochemical noise (EN)	EN describe the fluctuations of current and potential spontaneously generated by corrosion reactions.	Simple to use, no interference to the system, and measured signals can be analyzed by mathematical analysis.	The complicated kinds of noise (*i.e*., physical origin) due to corrosion of steel reinforcement make mathematical analysis unsuccessful.	Noise resistance (*Rn* (kΩ·cm^2^))	Electrodes (reference, counter, and working), Voltmeter, Ammeter, amplifier, and data acquisitions board.
**2**	**Elastic Wave Method**					
	Ultrasonic pulse velocity (UPV)	Mechanical energy propagates through the concrete as stress waves and is converted into electrical energy by a second transducer.	A large penetration depth and it is easy to use for estimating the size, shape and nature of the concrete damage.	The evaluation of UPV data is a highly specialized task, which requires careful data collection and expert analysis.	Pulse velocity (V)	Transducers (transmitter and receiver), amplifier, and oscillator.
	Acoustic emission (AE)	Elastic waves are generated due to rapid release of energy from a localized source within an RC structure.	A cost-effective and sensitive technique that can detect and locate the active defects.	Passive defects cannot be effectively detected.	AE parameters	Transducer, preamplifier, filter, amplifier, and storage equipment.
	Impact echo (IE)	Stress wave are propagated within the RC structure through vibrations and impact load.	A simple, fast, reliable method for inspecting the concrete is to impact the surface with a hammer and listen to the results.	The reliability of the IE method decreases with an increase in thickness.	Wave velocity (Vp)	Mechanical impactors, high-fidelity receiver, and data acquisition-signal analysis system.
**3**	**Electromagnetic Method**					
	Ground penetrating radar (GPR)	Transmission of electromagnetic (EM) waves into the RC structure under investigation.	Equipment portable and effective for investigating one large area from one surface.	Difficult interpretation of the results and needs post-processing analysis.	EM wave velocity (V)	Antennas (transmitter and receiver), a control unit, and computer.
**4**	**Optical Sensing Method**					
	Fiber Bragg grating (FBG)	The shift of FBG wavelength measures the increase in fiber strains with an increase in the cross section of steel reinforcement of corroded RC structures.	Small physical dimensions and suitable for embedding into structures.	The equipment has a high cost and there is no standardization of the procedure.	Bragg wavelength (λB)	Optical fiber sensor, Bragg meter, and computer.
**5**	**Infrared Thermography Method**					
	Infrared thermography (IRT)	IR radiation emitted by a concrete material is converted into an electrical signal and processed to create maps of the surface temperature.	Easy interpretation of the results and no radiation, rapid set-up, portable, and cost-effective technique.	There is no quantitative information on corrosion damage (*i.e*., size or depth).	Radiation power (E)	Multi spectrum camera.

## 3. Acoustic Emission (AE) for Corrosion Monitoring

The AE technique has been widely used in the field of civil engineering for structural health monitoring (SHM), especially the monitoring of corrosion [136,137,138]. The advantage of the AE technique is that can be used without intruding into any of the processes associated with the RC structures [139,140,141,142]. The first recorded application of the AE technique for corrosion monitoring was by Dunn *et al*. [143]. The AE technique was used to monitor and characterize the corrosion process in a series of controlled laboratory tests. The study illustrated the sensitivity of the AE technique to evaluate the ongoing corrosion process, suggesting its feasibility as a technique for monitoring corrosion in RC structures. There are two different approaches in analyzing AE data, one is the classical or parameter-based AE technique and the second is the quantitative or signal-based AE technique [142,144]. These approaches could be used for monitoring corrosion in RC structures, in the following sections.

### 3.1. AE Parameters for Corrosion Monitoring

**Figure 4:**
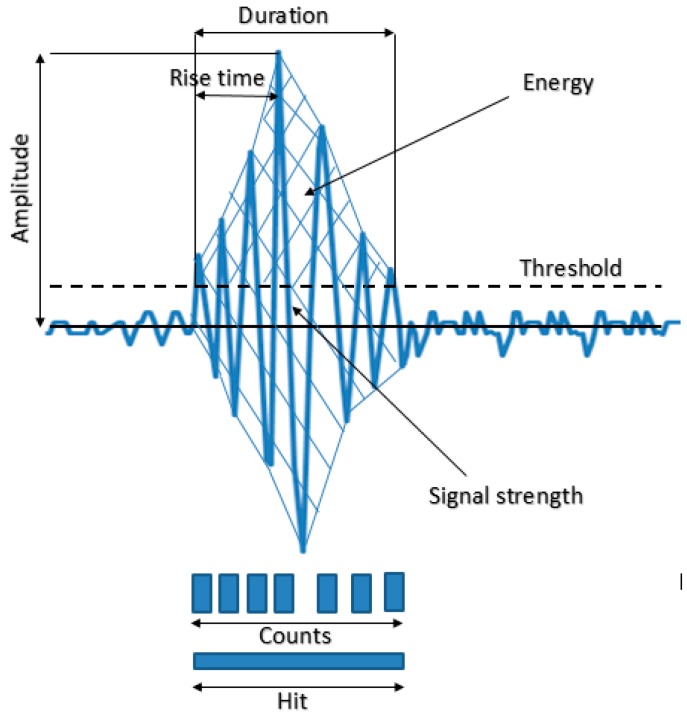
Parameters reflecting of an AE waveform.

The parameter-based technique is useful for better characterization of the AE source [145,146,147]. AE parameter analysis of hits or events, signal strength, and energy demonstrate that AE is readily applicable to the detection of corrosion in steel reinforcement, in order to identify the corrosion process, *i.e*., initiation of cracks and propagation of cracks, and to locate the early stages of corrosion [148,149,150,151,152,153,154,155,156,157]. The AE sources are also classified in terms of RA value and average frequency to classify the type of failure and the *b*-value or *Ib*-value of AE amplitude distribution for assessing the damage severity [151,153,158,159]. The above AE parameters will be reviewed in the following sections. A simplified representation of an emitted signal as well as of commonly used parameters is shown in Figure 4. A summary of AE parameters and their contribution in providing information about the source event are listed in Table 3. 

**Table 3:** AE parameters and the applications in source events information (Reproduced and modified from [158]).

Variables	Parameter	Description	Variables	Parameter
Time domain	Hit	Detection of an AE signal	Hit	Detection of an AE signal
	Event	Local material change, an event is a number of hits	Event	Local material change, an event is a number of hits
	Amplitude	Largest voltage peak in the waveforms	Amplitude	Largest voltage peak in the waveforms
	Rise time	Time elapsed from signal start and peak amplitude	Rise time	Time elapsed from signal start and peak amplitude
	Duration	Time between signal start and signal end	Duration	Time between signal start and signal end
	Threshold	Electronic compactor such that signals with amplitude larger than this level will be recorded	Threshold	Electronic compactor such that signals with amplitude larger than this level will be recorded
	Counts	Number of times AE signal crosses threshold	Counts	Number of times AE signal crosses threshold
	Signal Strength	Area under the positive and negative envelope of linear voltage signal	Signal Strength	Area under the positive and negative envelope of linear voltage signal
Frequency domain	Frequency spectrum	Nature of source event	Frequency domain	Frequency spectrum
Time-frequency domain	Spectrogram	Energy distribution of source event through time	Time-frequency domain	Spectrogram

#### 3.1.1. AE Hits

**Figure 5:**
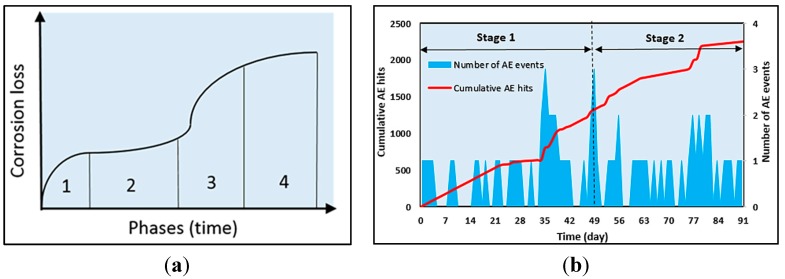
(**a**) Typical corrosion loss of steel reinforcement due to chloride immersion (Reproduced and modified from ([160]); and (**b**) cumulative AE hits and number of AE events during corrosion test (Reproduced and modified from [153]).

Many researchers have considered AE hits to be one of the AE parameters used to study the onset of corrosion and the nucleation of crack in RC structures [152,153,154,155]. Yoon *et al*. [152] carried out AE monitoring in RC beams subjected to four different degrees of corrosion. It was observed that AE hits increased with an increase in the degree of corrosion. This trend could provide important information for estimating the degree of corrosion. Ohtsu and Tomoda [155,156] investigated AE hits of RC specimens in sodium chloride (NaCl) concentration. Corresponding to two high AE activities, two periods of onset of corrosion and nucleation of cracks were observed. This could suggest that the AE activity observed corresponds to the corrosion loss of steel reinforcement in a marine environment observed by Melcher and Li [160], and shown in Figure 5a. At Phase 1, the onset of corrosion is initiated and the phase is dominated by the presence of oxygen and water. Then, corrosion loss decreases and stabilizes at Phase 2. The mass loss of corrosion increases again at Phases 3 and 4, where the corrosion penetrates inside and the expansion of corrosion products occurs due to anaerobic corrosion. Thus, based on the four phases of corrosion loss, two stages of corrosion activity are characterized, *i.e.*, the onset of corrosion and the growth of corrosion products (nucleation of cracks). 

On the other hand, Kawasaki *et al*. [153,154] showed cumulative AE hits and number of AE events in the corrosion process in a cyclic wet–dry test. The generating process of AE hits observed is classified into two stages, which refer to the process of corrosion loss of steel reinforcement due to chloride immersion as observed by Melcher and Li [160], and previously by Ohtsu and Tomoda [155,156]. Stage 1 (onset of corrosion) corresponds to Phases 1 and 2 and Stage 2 (nucleation of crack) corresponds to Phases 3 and 4. Thus, the AE technique could detect corrosion at an early stage and AE activities from the beginning, as in Figure 5b.

#### 3.1.2. Signal Strength (SS) and Cumulative Signal Strength (CSS)

Signal strength (SS) is one of the AE parameters; it is defined as the area under the voltage signal of AE over the duration of the waveforms. Since it provides a measure of the waveform energy released by the specimen, it is a rational damage indicator [67,161]. Velez *et al*. [161] showed the results of plots of the SS and cumulative signal strength (CSS) of corroded specimens. The SS of the prestressed specimen (P1) is attributed to the nucleation of cracks caused by the accumulation of corrosion products at the steel-concrete interface [162]. A different analysis is conducted on the SS of the non-prestressed specimen (N3), which suggests that it could be an indicator of early crack formation due to corrosion. On the other hand, the results show that the cumulative signal strength (CSS) exhibits a clear rate change before the onset of corrosion according to electrochemical methods, which suggests that the AE technique could detect the onset of corrosion.

**Figure 6:**
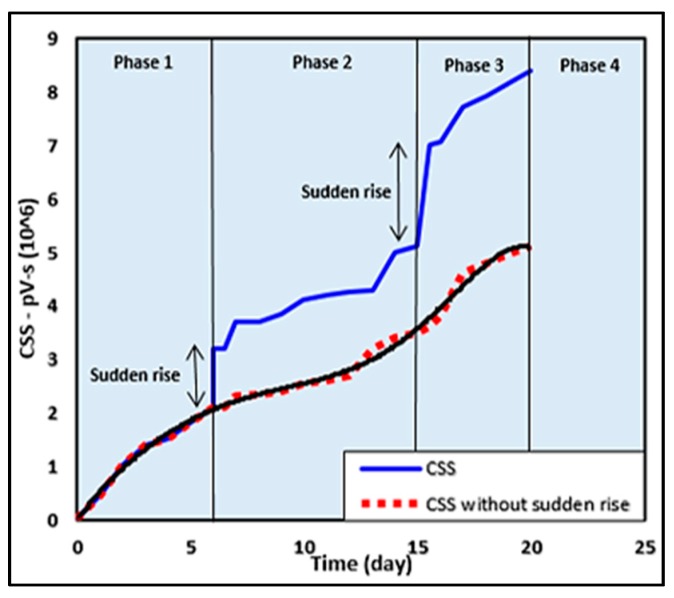
The variations in CSS parameter, which are similar to the curve of typical corrosion of steel reinforcement (Reproduced and modified from [68]).

Patil *et al*. [67] showed the curve of CSS with time of a concrete specimen in Figure 6. The curve clearly distinguishes the AE activity recorded for the concrete specimen under active corrosion. The CSS rate increases slowly in Phase 1 indicating de-passivation of the layer surrounding the steel reinforcement and the onset of corrosion. The presence of a sudden rise at the end of Phase 1 might indicate crack initiation due to steel corrosion. The rise of CSS in Phase 2 indicates corrosion activity. The sudden rise at the end of Phase 2 seems to be crack propagation leading to a macro-crack. Further phases are a repetition of Phase 2 continuing in the same manner. According to the phenomenological model of corrosion loss in a marine environment, the CSS curve obtained could also be divided into four phases, but the curve shows two sudden rises at the end of Phases 1 and 2. If these sudden rises are excluded from the CSS curve, the curve shown by the dotted line will be obtained and if connected by a smooth line, the curve would be exactly in agreement with the conventional curve (as shown in Figure 5a).

#### 3.1.3. Absolute (ABS) Energy

The feature of ABS energy is a quantifiable measurement of energy obtained for all the AE events or hits. Ing *et al*. [163] demonstrated the potential of ABS energy for identifying corrosion at an early stage before any external signs (*i.e*., cracking) of corrosion occur. The thickness of the concrete cover is found to have a significant effect on ABS energy in the early stages of steel corrosion. An exponential relationship has been established between the compressive strength and ABS energy, which shows that AE detects the sudden release of micro-fractures in the RC structure. In addition, increasing the steel diameter due to corrosion is found to increase the ABS energy of AE data.

#### 3.1.4. RA Values and Average Frequency

The characteristics of AE signals are estimated using two parameters, RA value and average frequency. The RA value and average frequency are defined from the AE parameters, *i.e.*, rise time, maximum amplitude, counts, and duration [151,156], as shown in Equations (3) and (4).

RA value = Rise Time/Amplitude
(3)

Average frequency = Counts/Duration
(4)

**Figure 7:**
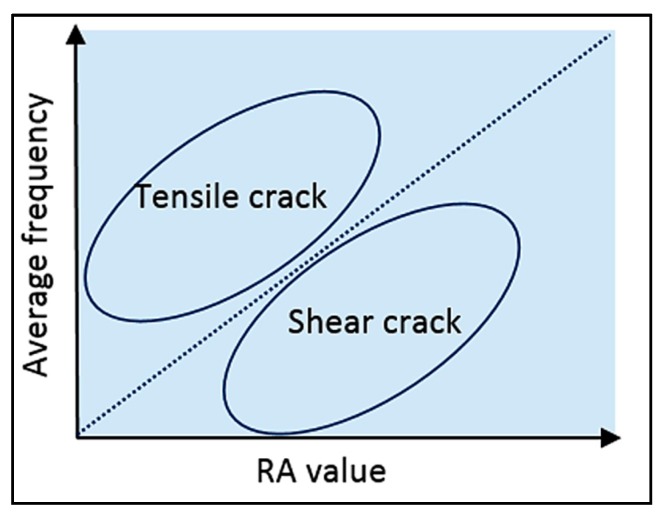
Classification of cracks by AE indexes (Reproduced and modified from [164]).

A crack type is classified by the relationship between RA value and average frequency as shown in Figure 7. A tensile-type crack is referred to as an AE signal with high average frequency and low RA value. A shear-type crack is identified by low average frequency and high RA value. This criterion is used to classify AE data detected in the corrosion process.

**Figure 8:**
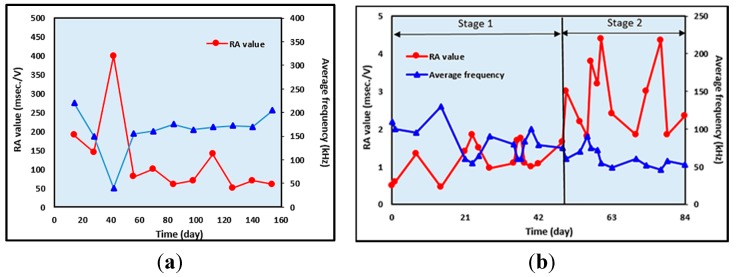
Variations in RA values and average frequency (**a**) Ohtsu and Tomoda **(**Reproduced and modified from [156]) and (**b**) (Reproduced and modified from Kawasaki *et al*. [153]).

The RA values and the average frequency were used by Ohtsu and Tomoda [156] to classify AE sources, in two weeks of wet and dry tests in an RC slab. At 40 days, the RA value was high and the AF was low, indicating shear cracking and later the RA value decreased and AF increased showing tensile cracking, as shown in Figure 8a. However, on the other hand, Kawasaki *et al*. [153] showed different trends for RA values and average frequency, as shown in Figure 8b. The trend lines proposed classify the onset of corrosion and nucleation of cracks in an RC beam. At Stage 1, at 14 days to 21 days, RA values drastically increase and the average frequencies become smaller. The crack could be classified as a tensile crack due to the onset of corrosion in the RC beam. At Stage 2, an increase in RA values and a decrease in average frequencies were observed. This implies that generations of nucleation of crack due to corrosion were induced in the concrete specimen. From the above references, the trends for RA value and average frequency, which were proposed by Ohtsu and Tomoda [156] for an RC slab, seem similar to the trends for RA value and average frequency proposed by Kawasaki *et al*. [153] for an RC beam at Stage 1. The RA value is low and the average frequency is fairly high.

#### 3.1.5. *b*-Value and *Ib*-Value

Gutenberg and Richter have developed the *b*-value in seismology to understand the relationship between the magnitude and frequency of earthquakes [140,165,166], as shown in Equation (5):

Log_10_ N = a−bM (5)

where, M is the Richter magnitude of the event, N is the incremental frequency, “*a*” is an empirical constant and *b* is the *b*-value. The value of M is proportional to the logarithm of the maximum amplitude A_max_ recorded in a seismic trace. 

In the AE method, the same principle can be applied to determine the scaling of the amplitude distribution of the AE waves during the fracture process. In term of AE technique, the formula was modified by Colombo *et al*. [140]. Where, A_max_ is peak amplitude of the AE events in decibels (dB).

Log_10_ N = a−b(Amax) (6)

However, to evaluate the slope failure and facture process, Shiotani *et al*. [167] improved the formula to improve b-value (*Ib*-value). This formulation is more based on statistical analysis, such as mean and standard deviation, for each of the AE amplitude sets. The formula is defined as:

(7) $$Ib=\;\frac{Log\;N\;\left(\mu -\;\alpha 1\sigma \right)-Log\;N\;\left(\mu -\alpha 2\sigma \right)}{\left(\alpha 1+\;\alpha 2\right)\sigma }$$

where, *σ* is the standard deviation, *μ* is the mean value of the amplitude distribution, *α*1 is the coefficient related to the smaller amplitude, and *α*2 is coefficient related to the fracture level. 

**Figure 9:**
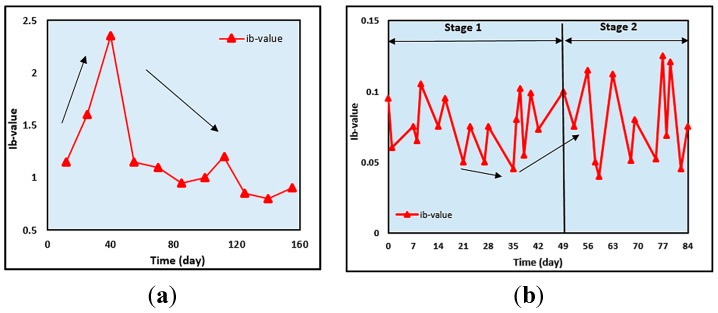
(**a**) *b*-value analysis (Reproduced and modified from [156]); and (**b**) *Ib*-value analysis (Reproduced and modified from [153]).

In an RC structure application, the *b*-value is used as an indication and demarcation of degradation in the integrity of the RC specimen and is associated with cracks [140]. When cracks are forming, the large number of events increases causing a decrease in the *b*-value. When the distributed micro-crack occurs at an early stage of corrosion, the *b*-value is large and when the macro-crack begins to localize the *b*-value is small. Previous research has indicated that *b*-values below 1.0 correspond to the transition from micro-crack to macro-crack [168,169]. Figure 9a shows an example of *b*-value distribution in acyclic wet–dry test in an RC slab by Ohtsu and Tomoda [156]. The *b*-value becomes large in the first period and then the *b*-values keep fairly low. This result might imply the generation of small shear cracks in the first period. Then, nucleation of fairly large tensile cracks follows, leading to the second period.

For the latter, the *Ib*-value is adopted for calculation, based on cumulative distribution as proposed by Shiotani [144]. In contrast, the case where the *Ib*-values become small implies nucleation of large AE hits. The variations in the *Ib*-value are given by Kawasaki *et al*. [153], as shown in Figure 9b. Large drops are observed between 21 days and 35 days, before the first dramatic increase in *Ib*-value. This might imply that the micro-cracks are generated at the onset of corrosion on the surface of the steel reinforcement. Due to high AE activity at Stage 2, the *Ib*-values decrease. Since the results of *Ib*-values at 56 and 84 days are comparatively lower than those of Stage 1, large-scale cracks are considered to be actively generated as corrosion-induced cracks in the RC beam. Furthermore, the fluctuations in *Ib*-values in Stage 2 are even bigger than in Stage 1. These results imply that the cracks are repeatedly generated due to corrosion products expansion.

#### 3.1.6. Intensity Analysis

Intensity analysis (IA) is used to quantify corrosion rate and level [170,171,172,173]. Intensity analysis evaluates the structural significance of an AE event by calculating two values, called the historic index (*Hi*) and the severity index (*Sr*), from the signal strength [174,175,176], as shown in Equations (8) and (9). The historic index is used to estimate changes of the slope in the CSS plotted as a function of time. The severity index is the average of the large signal strength received at a sensor (*i.e*., 50 events having the highest signal strength) [177]. An increase in severity index often corresponds to structural or material damage.

(8) $$Hi=\;\frac{N}{N-K}+\;\left(\frac{\sum_{i=k+1}^{N}Soi}{\sum_{n=1}^{N}Soi}\right)$$

(9) $$Sr=\;\frac{1}{J}\;\left(\overset{J}{\underset{m=1}{\sum }}Som\right)$$

where, Hi = Historic index, N = Number of hits up to time t, S_oi_ = Signal strength of the *i*th hit, K = empirically derived constant based on material, Sr = Severity index, J = empirically derived constant based on material, S_om_ = signal strength of the *m*th hit where the order of m is based on magnitude of the signal strength. 

The K and J value are related to N by the relations: K = 0, N ≤ 50; K = N−30, 51 ≤ N ≤ 200; K = O.85N, 201 ≤ N ≤ 500 and J = 0, N < 50; J = 50, J ≥ 50.

**Figure 10:**
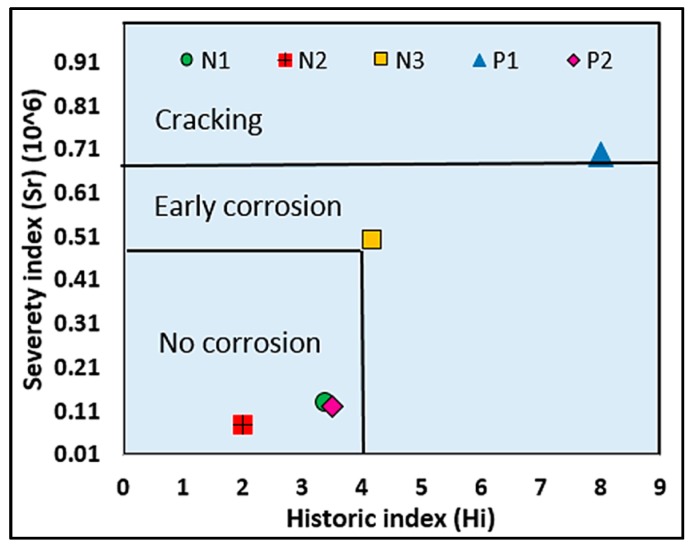
Intensity analysis results (Reproduced and modified from [161]).

Velez *et al*. [161] developed IA-based criteria for assessing corrosion in prestressed concrete (PC) piles. An assessment chart divides the criteria into three areas, *i.e*., no corrosion, early corrosion, and cracking, as shown in Figure 10. The figure shows that corroding and non-corroding specimens can be distinguished. In addition, the values of *Hi* and *Sr* are consistent with the levels of corrosion. 

#### 3.1.7. Relaxation Ratio

This refers to the ratio of average energy recorded during the unloading and loading phases of a concrete test [177]. The method is based on the effect of the cracks present in RC structures. If the RC structure contains no cracks, the energy recorded during the unloading phase is low, however, if the cracks exist, the cracks emit significant energy during the unloading phase. Therefore, the greater amount of AE energy collected during the unloading phase compared to the loading phase could be used as an indicator of corrosion, if the relaxation ratio is more than 1 [159].

### 3.2. Signal-Based AE for Corrosion Monitoring

The signal-based technique involved a large number of waveforms that are recorded over a sufficiently short period time [142,144]. The most prominent feature of this approach compared to parametric analysis is that it performs better in filtering signal noise, thus offering a better interpretation of the data monitoring in RC structures [158].

#### 3.2.1. AE Source Location

**Figure 11:**
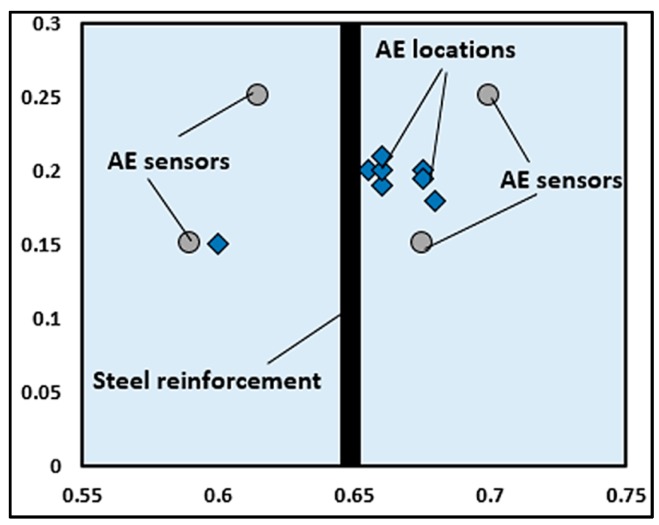
AE source location of corroded RC slab, unit (m) (Reproduced and modified from [155]).

AE source location is performed to monitor the onset of corrosion, crack initiation due to corrosion, crack propagation, and location of corrosion in RC structures. The AE source location is determined using the velocity of the longitudinal wave computed by the time differences among the arrival times of the first longitudinal wave detected by AE sensor [178]. Figure 11 shows the AE source location of high corrosion activity in an RC slab by Ohtsu and Tomoda [155]. It is reasonable to assume that AE sources are located around the steel reinforcement. This implies that corrosion and activities *i.e.*, cracks and the expansion of corrosion products are readily detected and located by the AE technique.

#### 3.2.2. SiGMA

**Figure 12:**
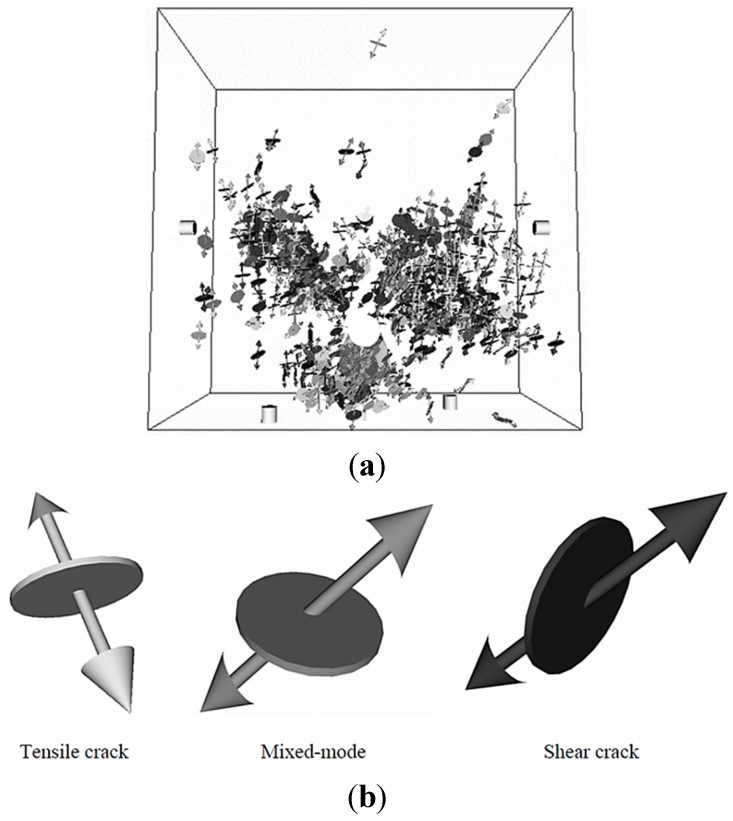
Results and models of SiGMA data (**a**) 594 events; and (**b**) models of tensile, mixed-mode, and shear crack (Reproduced and modified from [181]).

In order to determine the moment tensor of an AE source, a simplified procedure has been developed and implemented as SiGMA (Simplified Green’s functions for Moment tensor Analysis) by Ohtsu [179]. The analysis consists of a three-dimensional (3D) AE source location procedure and moment tensor analysis of AE sources. The location of the AE source is determined from the differences in arrival time [178]. Then, the components of the moment tensor are determined from the amplitudes of the first motions at the AE channels [153]. For this matter, SiGMA is a sophisticated method for estimating the size, orientation, crack type, location, and fracture mode of individual micro-cracking [180]. Farid Uddin and Ohtsu [181] and Kawasaki *et al*. [153] presented the results of SiGMA at corrosion test in an RC structures. By SiGMA, the cracking mechanisms due to corrosion of 594 events were identified close to steel reinforcement by [181], as shown in Figure 12. The legend of SiGMA results is shown in Figure 12b. In other hand, Thirty AE events of cracking mechanisms are detected in Stage 1 by [153]. These events are located around the steel reinforcement and at around the top side of the RC beam. This phenomenon could be related to the corrosion initiation as shear and mixed-mode cracks. In Stage 2, 19 AE events were determined close to the steel reinforcement with those of corrosion-induced crack in concrete and related to tensile cracks.

#### 3.2.3. Noise Filtering

**Figure 13:**
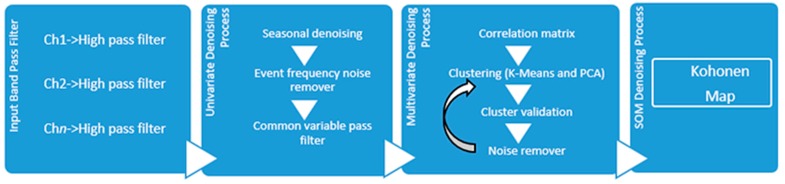
AE noise filtering procedure (Reproduced and modified from [183]).

The noise of AE data can originate from external sources, *i.e*., environment, traffic, man activities, *etc*. or be due to instrumental sources. Instrumental or electrical noise is generated by fluctuations occurring in the instrumentation including thermal noise, leakage current instability, and power supply voltage fluctuation [182]. Consequently, it is necessary to distinguish between real events related to real sources (such as crack propagation or fracture phenomena) and spurious events related to noise sources. Kouroussis and Anastassopoulos [183] proposed Unsupervised Pattern Recognition (UPR) to discriminate the signal and to distinguish real events from noise. The UPR applies mathematical or clustering algorithms in order to divide the set of AE hits into groups or clusters, which are close to one another in the same data set. This algorithm can discriminate the various sources of data that are related to the noise. Thus, Calabrese *et al*. [184] carried out an investigation to detect corrosion induced cracking in PC structures. It was focused on the use of multivariate analysis with the aim of establishing a procedure that would allow differentiation between steel corrosion based signals and background noise. Figure 13 shows the scheme for clustering methodology for noise removal. The three step models were implemented using Matlab software. The results show that more than 60% of detected signals were classified as noise.

## 4. Conclusions

A number of NDT methods for the corrosion monitoring of steel reinforcement have been reviewed in this paper. There are six major NDT methods for corrosion monitoring: *i.e*., visual inspection, electrochemical methods (*i.e*., HCP, resistivity method, LRP, and GPM), elastic wave methods (*i.e*., UPV, AE, and IE), the electromagnetic (EM) method (*i.e*., GPR), the optical sensing method (*i.e*., FBG), and the IRT method. NDT methods for corrosion evaluation are summarized in Table 2. Each technique was reviewed in relation to principles, certain applications, and limitations. However, AE is more effective for monitoring and detecting steel corrosion in RC structures at an early stage.

1. The early number of cumulative AE hits can detect the corrosion at early stage. If there is significant increase of cumulative AE hits, it corresponds to onset of corrosion.
2. Other AE parameters, like the sudden rise in cumulative signal strength (CSS) and absolute energy (ABS) might indicate crack initiation due to steel corrosion.
3. The distribution of RA value and average frequency (AF) are also proposed as a means of classifying the onset of corrosion and nucleation of cracks. The onset of corrosion is identified by a drastic increase in RA value and a decrease in average frequency. In addition, the nucleation of cracks is implied by an increase in RA value and a decrease in average frequency at the next stage. Based on the above parameters, the steel corrosion of RC structures can be analyzed according to two stages, *i.e.*, the onset of corrosion and the nucleation of crack due to corrosion product.
4. In addition, *b*-value, *Ib*-value, and intensity analysis are developed to characterize damage in RC structures. Previous studies have indicated that a *b-*value below 1.0 indicates the transition from microcracking to macrocracking. The large fluctuations in *Ib*-value imply that these cracks are generally repeated due to expansion of corrosion product. On the other hand, intensity analysis uses several criteria to identify the condition of the RC structure (*i.e*., no corrosion, early corrosion, and cracking).

Therefore, these capabilities make AE a strong candidate to become an efficient NDT method in the detection of corrosion occurring in real-time, giving it an advantage over other NDT methods. Although more research may be required to fully exploit current methods and devices, the future performance of AE in the corrosion evaluation of RC structures seems promising.
